# Marketing of nicotinamide as nicotine replacement in electronic cigarettes and smokeless tobacco

**DOI:** 10.18332/tpc/187767

**Published:** 2024-08-10

**Authors:** Sairam V. Jabba, Sven E. Jordt

**Affiliations:** 1 Center for Translational Pain Medicine, Department of Anesthesiology, Duke University School of Medicine, Durham, NC, United States; 2 Cancer Prevention and Control, Duke Cancer Institute, Duke University School of Medicine, Durham, North Carolina, United States; 3 Yale Center for the Study of Tobacco Products, Department of Psychiatry, Yale School of Medicine, New Haven, United States

**Keywords:** nicotinamide, 6-methyl nicotine, PMTA, non-cigarette tobacco products, tobacco industry, nicotine, nicotinic receptors

## Abstract

In the United States, the Food and Drug Administration (FDA) requires tobacco product manufacturers to submit Premarket Tobacco Product Applications (PMTA) for new products, granting marketing approval only if deemed appropriate for the protection of public health. Historically, the tobacco industry has exploited loopholes in the Tobacco Control Act (TCA), especially related to the definitions of nicotine, tobacco product and characterizing flavors, to circumvent the PMTA requirement. In 2023, the industry introduced several ‘PMTA-exempt’ e-cigarette and smokeless products, including products containing 6-methyl nicotine, a synthetic nicotine analog that is pharmacologically more potent than nicotine. In late 2023 and early 2024, the major US e-cigarette suppliers Nicotine River and ECBlend introduced ‘PMTA-exempt’ products with the brand names ‘Nixamide’ or ‘Nixodine’ or ‘Nixotine’, with nicotinamide as the main active ingredient. Nicotinamide is a form of vitamin B3 with no known pharmacological activity at nicotinic receptors. Here, we report that the marketing claims for these products, suggesting them and be nicotine substitute products designed to target nicotinic receptors and provide the same experience as nicotine, is deceptive and misleading to consumers. We also inform that these products have evolved further to contain a combination of nicotinamide and 6-methyl nicotine. The regulatory implications of these newly introduced products are discussed.

## INTRODUCTION

In the United States, tobacco product manufacturers are required to submit extensive Premarket Tobacco Product Applications (PMTA) for new products that undergo review by the FDA, resulting in either marketing approval or denial^1^. Unfortunately, the tobacco industry has repeatedly introduced products marketed as ‘PMTA-exempt’, exploiting loopholes in the Tobacco Control Act (TCA) and associated FDA regulations created by narrow or unclear statutory definitions of the terms ‘nicotine’ and ‘tobacco product’. One such example was the introduction of electronic cigarettes containing synthetic nicotine and not tobacco-derived nicotine. United States legislators quickly responded, amending the Tobacco Control Act in March of 2022 to give FDA regulatory authority over both natural and synthetic nicotine-containing products, with PMTAs required for both^2^. A more recent example is the introduction of an electronic cigarette product containing a structural nicotine analog, 6-methyl nicotine (6MN), that has similar or more potent pharmacological effects as nicotine at nicotinic receptors^3^.

## COMMENTARY

### Nicotinamide is marketed as a nicotine substitute for e-cigarettes

In October 2023, the major e-cigarette supplies wholesaler, Nicotine River, known for sales of bulk nicotine, solvents, and flavors, began sales of a nicotine substitute with the brand name ‘Nixamide’, a name trademarked by the company Ready Mix Naturals, LLC, and ‘with the main active ingredient being Nicotinamide’^4^. Nicotinamide, also named niacinamide, is a form of vitamin B3 and is commonly used in dietary supplements and medication to treat pellagra, a disease caused by the severe vitamin B3 deficiency, with symptoms of dermatitis, dementia, diarrhea and mouth sores. Nicotine River’s website states that Nixamide: ‘used in a vaping device, has been specially formulated to deliver similar satisfaction, pleasure, and enjoyment as traditional tobacco products and nicotine e-cigarettes for adult consumers. This product does not fall under the TCA (Tobacco Control Act)’^4^. In early 2024, nicotinamide-containing e-cigarette liquid became available for ordering by consumers from ECBlend, a major e-cigarette liquid vendor. These liquids are sold under the brand name ‘Nixotine^®^ flavored Nixodine^®^’ and are trademarked to Ready Mix Naturals and ECBlend LLC. Similar to Nixamide^™^, Nixotine^®^ flavored Nixodine^®^ is marketed to be: ‘formulated using a proprietary blend with the main active ingredient being Nicotinamide’^5^. ECBlend’s marketing material states: ‘Nixotine provides the same great sense of satisfaction, pleasure, and enjoyment as nicotine, it is not made or derived from tobacco or nicotine and does not consist of or contain nicotine from any source. Nixotine is not intended to be mixed with nicotine or other tobacco products’^5^. These statements suggest that nixotine has potentially nicotine-like pharmacological and behavioral actions. This perception is further strengthened by additional marketing statements, including: ‘Nixodine is carefully designed to target the same nicotinic acetylcholine receptors that traditional nicotine stimulates’^5^. Similar to their nicotine-containing e-liquids, ECBlend offers Nixotine^®^ in varying strengths from 3 mg to 36 mg. To our knowledge, no published reports have demonstrated that nicotinamide is either a nicotinic acetylcholine receptor (nAChR) agonist or is metabolized to form nicotine. Further, in rodent studies, nicotinamide administration was demonstrated to have sedative effects, in contrast to nicotine’s stimulatory effects. Nicotinamide has also been implicated in modifying drug-seeking behavior in rodents, where chronic nicotinamide administration throughout extinction reduced cocaine reinstatement in rats, whereas nicotine increased such behaviors. Interestingly, nicotinamide doses used in these studies were also several-fold higher than nicotine’s median lethal dose (LD50), suggesting that nicotinamide lacks stimulatory and toxic effects through nAChRs^6-9^. Claiming that a nicotinamide-based Nixotine product has the same molecular target as nicotine is either intentionally misleading, or the company may have added a nicotine analog with activity at nAChRs. The latter seems to be the case, as Nicotine River updated its website in April 2024 to reflect that Nixodine is a combination of nicotinamide and the nicotine analog 6MN. The website also lists products that contain only 6MN (Nixodine-S) and salt versions of nixodine and nixodine-S^10^.

An additional internet search revealed that nicotinamide is also added to smokeless products such as snuff and dip^11^, with the same trademarks (Nixodine^®^ or Nixamide^™^), marketing materials, and claims. The vendor emphasizes that nicotinamide has GRAS (‘Generally Recognized as Safe’) status, an FDA safety designation for food additives. A search of community forums revealed that some vape shops are sending marketing materials to their customers advertising new e-cigarette liquids that contain Nixodine, suggestive of a concerted marketing campaign supporting nicotinamide products^12^.

### Regulatory implications

The introduction of nicotinamide as a nicotine replacement in e-cigarette and smokeless products represents a significant regulatory challenge for the FDA. Nicotinamide does not have nicotinic receptor agonist activity and is known to act as a sedative at high dosages^6^. The claims made by Nicotine River, ECBlend, and other vendors that their product ‘provides the same great sense of satisfaction, pleasure and enjoyment as nicotine’ and ‘Nixotine^®^ may be addictive’ or ‘Nixodine is carefully designed to target the same nicotinic acetylcholine receptors that traditional nicotine stimulates’ suggest that these products are either marketed with inaccurate information or do contain an additional nicotinic receptor agonist. Though the vendor website is now updated to indicate that Nixodine/Nixotine contains 6MN, a nicotine analog that activates nAChRs, the exact amounts of 6MN added to the product is not provided on the product label^10^. Further, marketing material for Nixotine, states that the ‘main active ingredient’ is nicotinamide with no mention of 6MN^5^. Either way, the products and their marketing are clearly designed to circumvent FDA’s PMTA process, rejecting regulatory oversight and saving the effort and expense required for filing PMTAs. The exact composition of these products and amounts of the active ingredients need to be determined to enable the FDA to assess their regulatory status and risk assessment. FDA needs to also assess whether the agency is authorized to regulate the presence of nicotine analog under the tobacco product category, or if legislators need to revise the Tobacco Control Act (TCA) to enable regulation. While the manufacturer claims that Nixotine products do not fall under TCA, it is not up to the manufacturer to make such statements. FDA needs to review these products and decide whether such products fall under the TCA, under the Supplements Act, or require regulation as drugs. Further, the TCA gives the FDA the authority to regulate products that are marketed with deceptive and misleading information to consumers^13^.

Historically, from the 1970s, the tobacco industry has conducted pharmacological studies on several nicotine analogs, to understand their pharmacological effects and determine their potential to replace nicotine in products and also to circumvent any foreseeable regulations on nicotine^14^. Earlier receptor binding and rodent studies have demonstrated that, compared to nicotine, several methylated nicotine analogs, including 6MN, have differential pharmacological, behavioral, and toxicological effects. 6MN was 3-fold more potent in binding to nAChRs and more toxic (1.5-to 3-fold lower LD50 dose)^3,15,16^. These analogs have never been commercialized and brought to the market until now. However, with the FDA introducing more stringent regulations to obtain marketing authorization for new tobacco products, the marketplace is witnessing the introduction of these nicotine analog-containing products with claims of being ‘PMTA exempt’ to circumvent these regulations^3^.

For those vaping products that do not contain nicotine or nicotine analogs but only nicotinamide, a vitamin B3 analog, these companies should not be permitted to continue advertising that their product has nicotinic acetylcholine receptor activity. Vendors may claim that their nicotinamide-containing products should be regulated as foods or supplements, with nicotinamide designated as GRAS as a food additive. However, a GRAS designation does only apply for the conditions of the intended use scenario as a food additive and does not apply to inhalational intake through an e-cigarette^17^. While inhalation toxicity data for such a scenario are not available^18^, the chemical safety documentation materials warn that exposure to nicotinamide powder may cause respiratory irritation, recommending to avoid formation of dust and aerosols^18^. The inhalation of vitamins is not without risk. For example, the illegal and unauthorized use of vitamin derivatives such as Vitamin-E acetate in vaping products has led to fatal e-cigarette or vaping product use-associated lung injury (EVALI) outbreaks^19^.

Vitamins and hormone-containing wellness vapes have become popular in the US and globally and are often marketed with misleading information^20,21^. Recently, the FDA has issued warning letters to manufacturers of several vitamin vaping products for marketing their products with unproven health claims and due to safety and toxicity concerns, especially for those containing vitamin B12^22,23^. But unfortunately, in the US, these vitamin products remain largely unregulated by the FDA. Unlike in the US, such non-nicotine or nicotine-free vapes are regulated in several EU countries, including Germany, Belgium, and the Netherlands, where they are subject to the European Tobacco Product Directive (TPD)^24^. As per the TPD, vitamins and additives that create an impression that the e-cigarette has beneficial health effects are not allowed to be added to vaping products^24^. As the manufacturers of these nicotinamide vape products assert that nicotinamide is the ‘main active ingredient’ but also claim to have pharmacological activity at nAChRs, the FDA can alternatively categorize these products as a drug and regulate them under the Federal Food, Drug, and Cosmetic Act (FDCA). Either way, if the FDA is not provided with the regulatory tools to take appropriate enforcement action against these new and emerging products marketed as ‘PMTA Exempt’, the FDA’s authority and its capability to protect public health are further undermined.

## CONCLUSION

Nicotinamide- and 6-methyl nicotine-containing products, claimed to be nicotine replacements and ‘PMTA-exempt’, represent a significant regulatory challenge for FDA. The FDA needs to determine if these products fall under the purview of the TCA or need to be re-categorized as drugs and regulated under the Federal Food, Drug, and Cosmetic Act (FDCA). Either way, the FDA needs appropriate regulatory tools to enforce its authority over the rapidly evolving marketplace of nicotine substitute products, to protect public health.

## Data Availability

The data supporting this research are available from the authors on reasonable request.
